# Eocene sand tiger sharks (Lamniformes, Odontaspididae) from the Bolca Konservat-Lagerstätte, Italy: palaeobiology, palaeobiogeography and evolutionary significance

**DOI:** 10.1080/08912963.2017.1341503

**Published:** 2017-06-22

**Authors:** Giuseppe Marramà, Andrea Engelbrecht, Giorgio Carnevale, Jürgen Kriwet

**Affiliations:** a Department of Palaeontology, University of Vienna, Vienna, Austria; b Dipartimento di Scienze della Terra, Università di Torino, Torino, Italy

**Keywords:** Chondrichthyes, Elasmobranchii, *Brachycarcharias* Cappetta and Nolf, 2005, Ypresian, Konservat-Lagerstätte, biotic turnovers

## Abstract

Here we report the first record of one of the most common and widespread Palaeogene selachians, the sand tiger shark *Brachycarcharias*, from the Ypresian Bolca Konservat-Lagerstätte. The combination of dental character of the 15 isolated teeth collected from the Pesciara and Monte Postale sites (e.g. anterior teeth up to 25 mm with fairly low triangular cusp decreasing regularly in width; one to two pairs of well-developed lateral cusplets; root with broadly separated lobes; upper teeth with a cusp bent distally) supports their assignment to the odontaspidid *Brachycarcharias lerichei* (Casier, 1946), a species widely spread across the North Hemisphere during the early Palaeogene. The unambiguous first report of this lamniform shark in the Eocene Bolca Konservat-Lagerstätte improves our knowledge concerning the diversity and palaeobiology of the cartilaginous fishes of this palaeontological site, and provides new insights about the biotic turnovers that involved the high trophic levels of the marine settings after the end-Cretaceous extinction.

## Introduction

Extant selachians of the family Odontaspididae, also known as sand tiger sharks, include large size species (up to 4.5 m) within the order Lamniformes, that inhabit marine tropical to cold waters of the continental and insular shelves to deep slopes (up to 1600 m) of the Atlantic, Indian, and Pacific oceans (Compagno 1984; Cappetta 2012; Nelson et al. 2016; Froese and Pauly 2017). Although some recent morphological and molecular analyses suggest that Odontaspididae might be non-monophyletic (e.g. Shimada 2005; Martin et al. 2002; Naylor et al. 2012), several authors have recognised a set of morphological characters that are used traditionally to distinguish sand tiger sharks from all other lamniforms, including a short to moderately long, conical or slightly depressed snout, weakly protrusible jaws, first dorsal fin in front of the pelvic origin, last gill-slit in front of the pectoral origin, 156–183 vertebral centra, monognathic tooth heterodonty, tearing-type dentition, teeth arranged in less than 60 rows in each jaw having a tall and slender main cusp, one to three pairs of lateral cusplets, and a root with well-separated lobes and marked by a strong nutritive furrow (Compagno 1999; Cappetta 2012; Nelson et al. 2016). Today, odontaspidids are only represented by three species in the genera *Carcharias* and *Odontaspis* (Compagno 1999; Nelson et al. 2016). Although the odontaspidid fossil record has a wide temporal and geographic distribution, with at least 20 genera and more than 50 species dating back to the Lower Cretaceous (Cappetta 2012; Shimada et al. 2015; Cappetta and Case 2016), very little is known about how and to what extent global changes affected the biodiversity and evolutionary history of individual genera, notably those temporarily close to biological crisis events. Although general patterns of abiotic disruptions during the Palaeogene have been extensively documented (e.g. Culver and Rawson 2000) with the fossil record documenting profound changes in both marine and terrestrial ecosystems across the Palaeocene-Eocene Thermal Maximum (e.g. Gingerich 2006), cartilaginous fishes during this period have received little attention.

The world-famous Eocene (Ypresian, ca. 50 Ma; Papazzoni et al. 2014a) Bolca Konservat-Lagerstätte in Italy, is one of only three Palaeogene deposits (including the Eocene Green River Formation in Wyoming, and the Oligocene Grube Unterfeld in Germany; see de Carvalho et al. 2004; Hovestadt et al. 2010), in which chondrichthyans are exquisitely preserved. In addition to complete and well-articulated specimens, the fossils of Bolca also include isolated shark teeth, which are crucial to reconstruct the taxonomic diversity of chondrichthyans during the Eocene, and to better understand the biological shifts that occurred after the end-Cretaceous extinction and those that shaped the biotic configuration of modern marine ecosystems. The Bolca sites date back about 15 Ma after the end-Cretaceous extinction, corresponding to a period of maximum morphological diversification of major fish lineages (Friedman 2010; Near et al. 2013) and coincides chronologically with the latest phase of the Early Eocene Climatic Optimum (Papazzoni and Trevisani 2006; Papazzoni et al. 2017). Although several studies in the last four centuries contributed to our knowledge of the extraordinary palaeobiodiversity of this deposit, with more than 230 teleost species having been described so far (Carnevale et al. 2014), many aspects concerning the palaeobiodiversity, palaeoecology, and evolutionary significance of cartilaginous fishes have been neglected or underestimated up to now.

The goal of this paper is to provide the first unambiguous record of one of the most common and widespread early Palaeogene selachians, *Brachycarcharias* Cappetta and Nolf, 2005, in the Eocene Bolca Lagerstätte, the only elasmobranch from this deposit represented uniquely by isolated teeth. Palaeobiogeographic, palaeobiological and palaeoecological implications based on a comprehensive analysis of the fossil record of this genus, provide new insights into the biotic turnovers that occurred at high trophic level of the marine food chain during the Palaeogene.

## Geological setting

**Figure 1.**
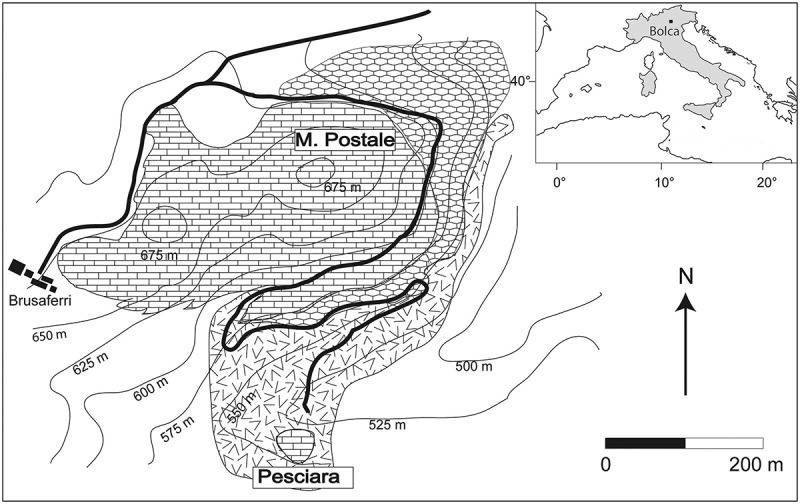
Location and geological map of the Bolca area showing the sites of Ypresian age where *Brachycarcharias lerichei* (Casier, 1946) teeth have been found.

Teeth were collected from the fossiliferous layers of the Pesciara and Monte Postale sites of the Bolca Konservat-Lagerstätte, located in the eastern part of the Lessini Mountains (southern Alps), about 2 km north-east of the village of Bolca, Verona Province, north-eastern Italy (Figure 1). These two sites are about 300 m from each other and exhibit similar stratigraphic and sedimentological features, mostly related to the presence of finely laminated micritic limestone with fish and plant remains. The stratigraphic relationships between the two fossiliferous deposits recently have been investigated by Papazzoni et al. (2017), who suggested that the uppermost fossiliferous sequence of Monte Postale and that of the Pesciara sites stratigraphically belong to the same biozone (SBZ 11, *Alveolina dainelli* Zone; Ypresian, middle Cuisian), although the fossiliferous limestones of the Pesciara appear to be slightly younger (Papazzoni et al. 2017). Controlled excavations from 1999 to 2011 conducted at both sites by the Museo Civico di Storia Naturale di Verona, provided a better understanding of their palaeoenvironmental settings (see Marramà et al. 2016a).

The stratigraphic sequence of the Pesciara site was investigated by several authors who referred the fossiliferous layers to the ‘Calcari Nummulitici’, an informal unit of Eocene age widely distributed in northeastern Italy (see Papazzoni and Trevisani 2006). The entire succession of the Pesciara site consists of a less than 20 m thick cyclic alternation of finely laminated micritic limestones, with exquisitely well-preserved fishes, plants and invertebrates, and coarse-grained biocalcarenite/biocalcirudite containing a rich benthic fauna. Based on their larger benthic foraminiferan content, the fish-bearing limestones of the Pesciara site were referred to the *Alveolina dainelli* Zone (Papazzoni et al. 2014a), corresponding to the late Cuisian (late Ypresian, slightly less than 50 Ma; Papazzoni and Trevisani 2006). Results of recent quantitative palaeoecological analysis by Marramà et al. (2016a) confirm that the Pesciara fish assemblage is defined by a sharp oligarchic structure dominated by zooplanktivorous fishes (mostly clupeoids; see also Marramà and Carnevale 2015a, 2015b, 2016), whereas the taphonomic features suggest that the sediments were deposited in an intraplatform basin in which benthic anoxic conditions and the development of a biofilm acted as promoters of the high-quality preservation of the fossils (see also Papazzoni and Trevisani 2006).

The Monte Postale succession includes the Cretaceous Scaglia Rossa Formation up to the Ypresian fossiliferous limestones. The first stratigraphic studies of the Monte Postale site by Fabiani (1914, 1915) assigned the entire succession to the Lutetian. Hottinger (1960) and more recently Papazzoni et al. (2017) suggested that the uppermost productive strata of the Monte Postale site are, conversely, Ypresian in age, based on the foraminiferal and calcareous nannofossil content. The palaeoecological and taphonomic study of the Monte Postale fish assemblage revealed a high fish diversity within a different depositional context with respect to that hypothesised for the Pesciara site (Marramà et al. 2016a). In fact, the abundance of marine and terrestrial plants, the large number of invertebrates (including abundant corals) and reef-associated small-sized and juvenile fishes at the Monte Postale site, suggest that the sediments accumulated close to an emerged coastal area (lagoon) surrounded by a coral reef. Moreover, the prominent disarticulation of fish skeletons, unimodal dispersion of elements, and bioturbations were probably the results of disturbance and at least periodic oxic conditions at the bottom (Marramà et al. 2016a; Vescogni et al. 2016).

## Material and methods

The present study is based on 15 isolated teeth from the fossiliferous layers of the Pesciara and Monte Postale sites. The fossils are currently housed in the collections of the Museo Civico di Storia Naturale di Verona (MCSNV), Museo dei Fossili di Bolca (technically part of the MCSNV), Massimo Cerato’s registered collection (MC), Museo di Geologia e Paleontologia dell’Università degli Studi di Padova (MGP-PD), and Natural History Museum of London (NHMUK). In addition to the two specimens from Monte Postale examined at the end of XIX century by Bassani (1897) (MGP-PD 7358; MGP-PD 7366; see Remarks), we examined new material including specimens collected in the second half of the XX century and those from the most recent controlled excavations carried out from 1999 to 2011 (see Marramà et al. 2016a). All the specimens are still embedded in the matrix since bureaucratic rules of the collection did not allow for the removal of the specimens from it. However, the specimens were mechanically prepared with needles to reveal fine details. The attempted micro-CT scan, performed in the lab of the Department of Palaeontology of the University of Vienna, did not reveal further details of the unexposed sides, since the similar density of the matrix and that of the teeth do not allow distinguishing the two components. Subsequently, all the teeth were assigned to their respective positions using characters observed in *Brachycarcharias lerichei* mostly following Cappetta and Nolf (2005), Van Den Eeckhaut and De Schutter (2009), Cappetta (2012), and Cappetta and Case (2016). Morphological tooth terminology follows Cappetta (2012). Morphometric terminology is adopted and modified from Kriwet et al. (2015) (see also Supplementary material).

Tooth measurements, taken to the nearest 0.01 mm, were used to provide additional support for the identification of the jaw position, and to provide a body size estimation of the individuals, which is necessary to infer palaeobiological and palaeoecological aspects. All the measurements were standardised for the height of the lateral cusplets (LCH, one of the measurements that was possible to detect in most of the specimens) to remove the size effect, and log-transformed in order to eliminate the variation due to ontogeny (allometric effect). A principal component analysis (PCA) was then performed using the software package PAST 3.11 (Hammer et al. 2001) on the entire data-set of standardised and log-transformed measurements in order to provide a direct visual image of the spatial separation of specimens, and to support the identification of their original jaw position. Moreover, we used the non-parametric multivariate analysis of variance (PERMANOVA; Anderson 2001) to provide statistical support to the separation of the groups.

The range of body sizes for *B. lerichei* in the Bolca palaeobiotope was estimated employing the method proposed by Shimada (2004), who examined the relationship between crown height (CH) for every tooth position in the living odontaspidid *Carcharias taurus*, and established equations to infer its total body length (TL). Since the largest tooth can be used as a proxy for the body size estimation in fossil sharks (Cappetta 2012), we used the equations for the largest upper antero-lateral (TL = −26.665 + 12.499 CH), upper lateral (TL = −9.355 + 17.708 CH), and lower anterior tooth (TL = −24.722 + 10.305 CH) of *C. taurus* in order to estimate the body size of all individuals in the Bolca palaeobiotopes. We used the odontaspidid *C. taurus* for the body size estimation of *B. lerichei*, since this species is the closest living relative for which the relationship tooth-body size is known.

## Systematic palaeontology

Class CHONDRICHTHYES Huxley, 1880

Subclass ELASMOBRANCHII Bonaparte, 1838

Order LAMNIFORMES Berg, 1958

Family ODONTASPIDIDAE Müller and Henle, 1839

Genus *Brachycarcharias* Cappetta and Nolf, 2005

*Type species*. *Lamna lerichei* Casier, 1946; Lower Eocene, Ypresian; Forest-lez-Bruxelles, Belgium

*Included species*. *B. atlasi* (Arambourg, 1952); *B. lerichei* (Casier, 1946); *B. koerti* (Stromer, 1910); *B. mississippiensis* (Case, 1994)

*Brachycarcharias lerichei* (Casier, 1946)

(Figures 2–3)

**Figure 2.**
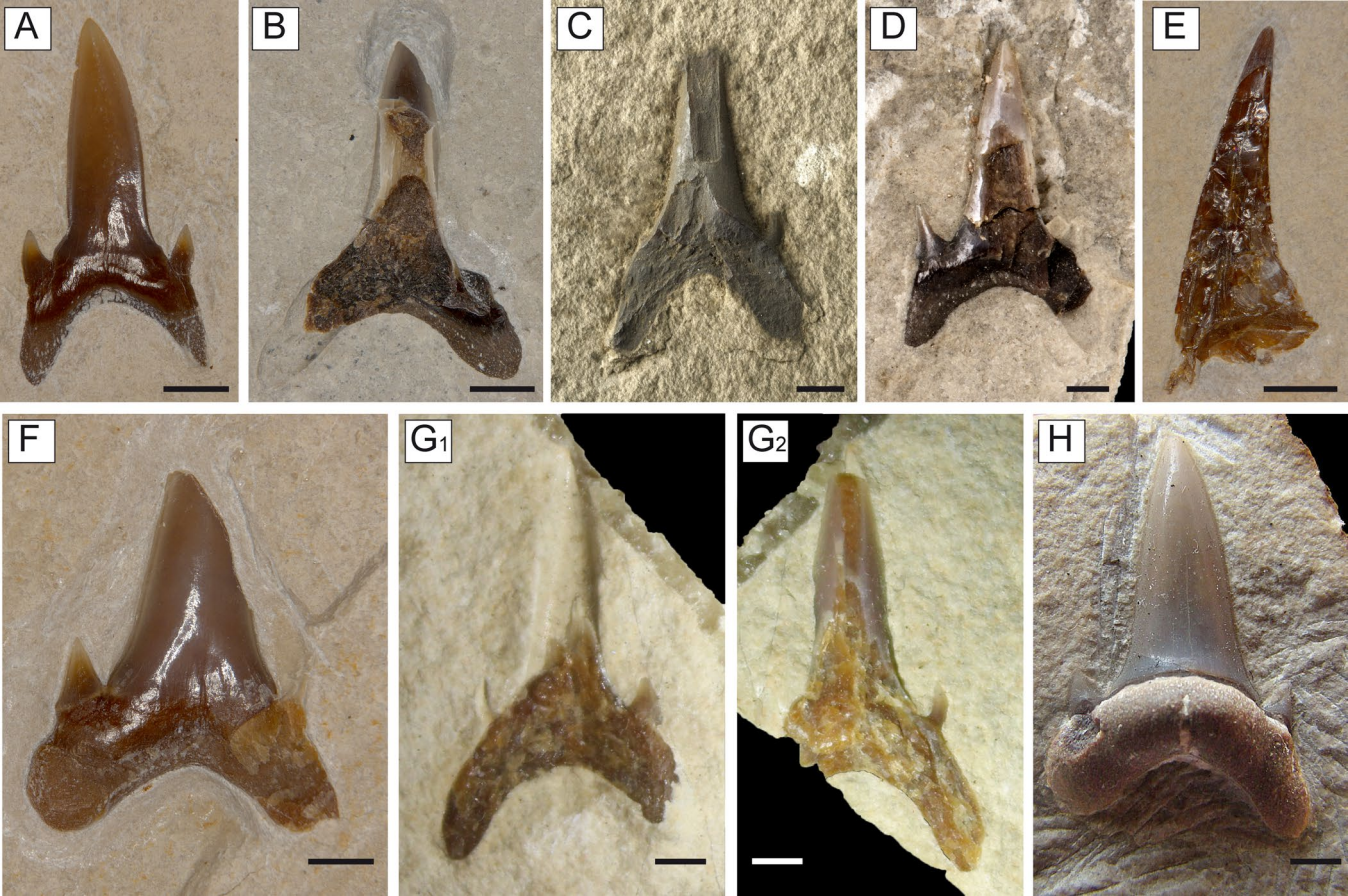
*Brachycarcharias lerichei* (Casier, 1946) from the Eocene Bolca Lagerstätte, Italy. (A–E) anterior teeth: (A) MCSNV IG.VR.69800, labial view, Monte Postale site; (B) MCSNV IG.VR.24423, labial view, Pesciara site; (C) MGP-PD 7358, lingual view, Monte Postale site, figured in Bassani (1897, pl. 9, fig. 12); (D) NHMUK PV.OR.43450, labial view, Pesciara site; copyright: The Trustees of the Natural History Museum, London; (E) MCSNV IG.VR.69757, profile view, Monte Postale site. (F–H) lower antero-lateral teeth: (F) MCSNV IG.VR.66977, labial view, Monte Postale site; (G_1_–G_2_) MCSNV IG.135777/8, specimen in part and counterpart, Pesciara site; (H) MCSNV IG.135779, lingual view, Pesciara site. Scale bars 2 mm.

**Figure 3.**
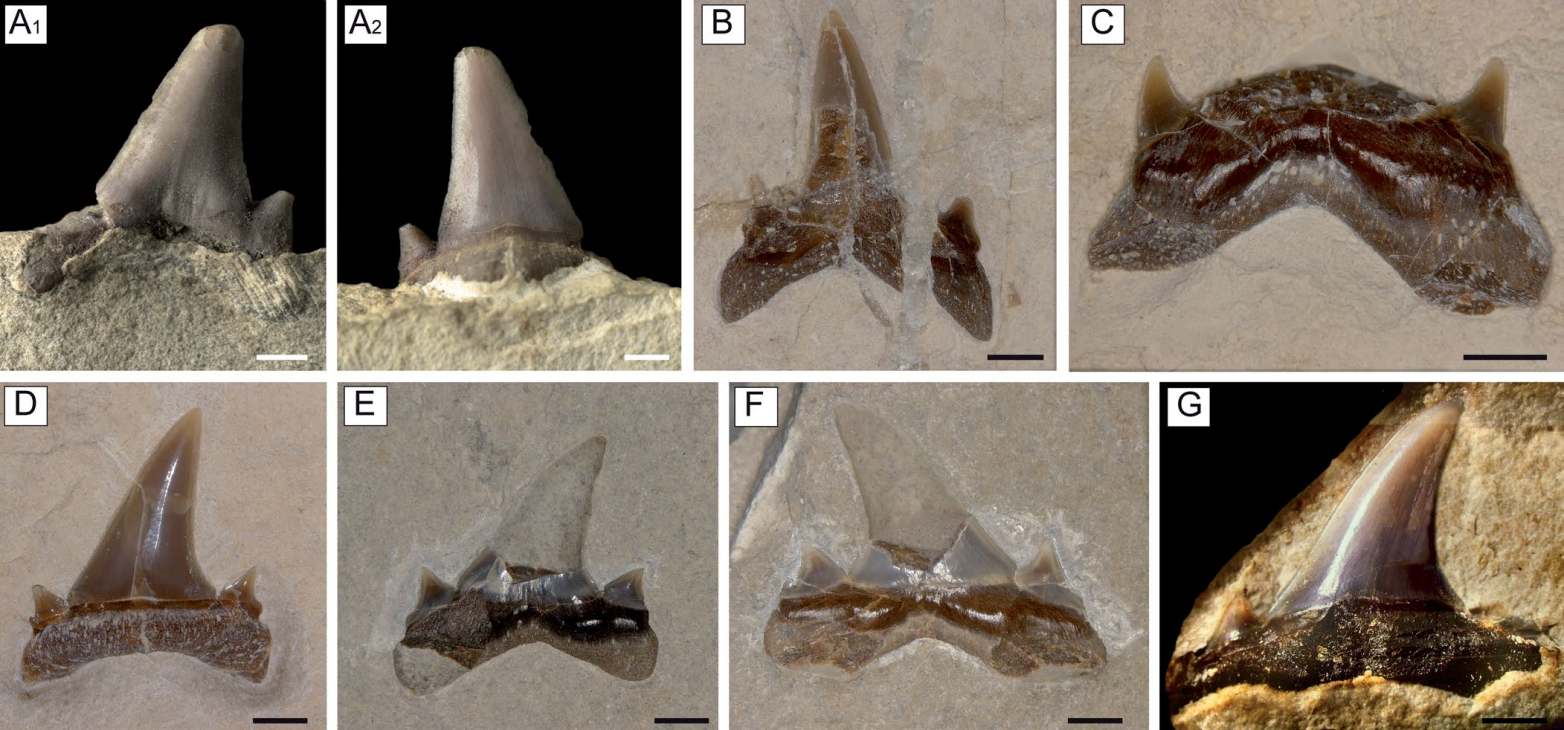
Upper teeth of *Brachycarcharias lerichei* (Casier, 1946) from the Eocene Bolca Lagerstätte, Italy. (A–C) antero-lateral teeth: (A_1_) labial and (A_2_) lingual view of MGP-PD 7366, Monte Postale site, figured in Bassani (1897, pl. 9, Fig. 11); (B) MC 89, labial view, Monte Postale site; (C) MCSNV IG.VR.24339, labial view, Monte Postale site; (D–G) lateral teeth: (D) MCSNV IG.VR.69484, lingual view, Monte Postale site; (E) MCSNV IG.23598, labial view, Pesciara site; (F) MCSNV T.176, labial view, Pesciara site; MCSNV IG.43355, lingual view, Pesciara site. Scale bars 2 mm.

See Cappetta and Nolf (2005) for a complete list of synonyms.

*Lamna vincenti* Winkler, 1874: Bassani 1897, p. 87, pl. 9, figures 11–13

*Lamna vincenti* Winkler, 1874: Eastman 1904, p. 27

*Lamna vincenti* Winkler, 1874: Eastman 1905, p. 352

*Lamna* sp.: Stromer 1905, p. 182

*Odontaspis hopei* Agassiz, 1843: D’Erasmo 1922, p. 31 (pro parte)

*Lamna lerichei* Casier, 1946: Casier 1946, p. 80, pl. 2, figure 7a–b [after *Lamna vincenti* Winkler, 1874, pars, in Leriche (1906)]

*Brachycarcharias lerichei* (Casier, 1946): Cappetta and Nolf 2005, p. 241, pl. 9

*Referred material*. Fifteen isolated teeth: MC 89; MCSNV IG.43355; MCSNV IG.135777/8; MCSNV IG.135779; MCSNV IG.23598; MCSNV IG.VR.24339; MCSNV IG.VR.24423; MCSNV IG.VR.66977; MCSNV IG.VR.69484; MCSNV IG.VR.69757/8; MCSNV IG.VR.69800; MCSNV T.176; MGP-PD 7358; MGP-PD 7366; NHMUK PV.OR.43450.

*Locality and Horizon*. Pesciara and Monte Postale sites, Bolca Konservat-Lagerstätte, Italy; early Eocene, late Ypresian, middle Cuisian, SBZ 11, *Alveolina dainelli* Zone (see Papazzoni and Trevisani 2006; Papazzoni et al. 2017).

*Remarks*. Sharks from the fossil fish-bearing strata of the Bolca Lagerstätte are mentioned in the literature at least by the end of the XVIII century (e.g. Volta 1796). They are currently represented by several complete articulated skeletons belonging to the Carcharhiniformes, and include the triakid *Galeorhinus cuvieri* (Agassiz, 1835) and the carcharhinid *Eogaleus bolcensis* Cappetta, 1975 (see Cappetta 1975; Fanti et al. 2016). According to the synoptic list of the Eocene chondrichthyans from Bolca provided by Blot (1980), the selachians also included a representative of the order Orectolobiformes. However, a unique specimen of the bamboo shark *Mesiteia emiliae*, described as a Bolca selachian by Jaekel (1894) actually does not come from this deposit. Cappetta (1980) demonstrated a Late Cretaceous age and a probable Lebanese origin for the specimen, based on the foraminiferal content and the presence of the clupeomorph *Armigatus brevissimus* on the slab. Furthermore, Blot (1980) did not consider the isolated odontaspidid teeth from the Monte Postale site previously described by Bassani (1897), and those recovered from subsequent excavations at both sites. Bassani (1897) described seven teeth referring them to *Lamna vincenti* Winkler, 1876 from Monte Postale site, and nine teeth as belonging to *Odontaspis hopei* Agassiz, 1843 from an indeterminate site of ‘Monte Bolca’. Eastman (1904, 1905), Stromer (1905), and D’Erasmo (1922) also indicated the presence of odontaspidid specimens from the Bolca Lagerstätte, although without providing any new or additional description.

The presence of *Lamna vincenti* Winkler, 1876 was reported in several other Ypresian deposits of Europe, North America, and Africa (e.g. Woodward 1899; Casier 1946; Arambourg 1952; Noubhani and Cappetta 1997). In a first revision of the Ypresian material from Belgium by Casier (1946), the specimens traditionally assigned to *L. vincenti* were reclassified as *Lamna lerichei*. In a later revision of the same material by Cappetta and Nolf (2005), all *L. lerichei* teeth were observed to be considerably morphologically different from those of any known odontaspidid or lamnid species. Thus, Cappetta and Nolf (2005) erected the odontaspidid taxon *Brachycarcharias lerichei*, in order to include *Lamna vincenti* and *L. lerichei*. However, Purdy and Francis (2007) questioned the validity of *Brachycarcharias* but no synonymy with previously described genera or species was provided according to Cappetta (2012). Based on the present study, the teeth described and figured by Bassani (1897, pl. 9, figures 11–13) as *Lamna vincenti* correspond perfectly to the diagnosis of the anterior and upper anterolateral teeth of *Brachycarcharias lerichei*. All the other teeth from the most recent excavations conducted at Pesciara and Monte Postale sites can be unquestionably referred to this species (see Description).

Teeth referred to *Odontaspis hopei* by Bassani (1897, pl. 9, figures 14–15) were not collected from the Pesciara or Monte Postale sites. They are morphologically identical to those historically extracted from another palaeontological site near the Bolca area, the Spilecco site, and seemingly were not embedded in matrix (isolated teeth from Pesciara and Monte Postale always are embedded in and strictly associated to the sediment; G.M. and Dr. Roberto Zorzin pers. obs.), suggesting that they actually come from the aforementioned deposit. This site is older, and has different lithological, sedimentological, stratigraphic and palaeoenvironmental features compared to those of the Pesciara and Monte Postale sites (Papazzoni et al. 2014b). It must be noted that the unclear term ‘Monte Bolca’, used by Bassani (1897) to refer to the site of origin of the *O. hopei* teeth, has been often used in the past by several authors to generally refer to the Bolca area (which actually includes the Pesciara, Monte Postale, Spilecco, Vegroni, Purga di Bolca and other minor sites), without any indication of the precise origin of the material. However, an analysis of the teeth from the Spilecco site (possibly belonging to *Hypotodus* or *Striatolamia*, pending further studies) is beyond the scope of this paper and these teeth are not considered in this study.

### Description

Tooth morphology and size are consistent with those described by Cappetta and Nolf (2005) and Cappetta (2012). Measurements are given in Table 1. The largest tooth is an upper antero-lateral element of about 19.5 mm total height (TH). Teeth belonging to different positions (upper and lower, anterior, antero-lateral and lateral) have different morphologies, suggesting that the dentition is monognatic heterodont and of tearing type (Figures 2 and 3).

**Table 1.** Tooth measurements (lengths in mm; angles in degree), with relative position and estimated body size for *Brachycarcharias lerichei* (Casier, 1946) from the Eocene Bolca Lagerstätte.

Specimen	Site	BCW	CH	DCL	DS	LCH	MCL	PCH	PCW	RA	RW	RH	TH	BCT	Position	Body size estimation (cm)
MC 89	M. POSTALE	10.63	10.88	11.62	6.11	3.01	12.94	9.28	5.63	120.97	12.93	4.38	15.26	–	Upper antero-lateral	109.3
MCSNV IG.135777/8	PESCIARA	8.02	12.75	13.28	1.02	2.56	13.33	11.71	5.22	101.1	10.68	5.81	18.56	–	Lower antero-lateral	106.7
MCSNV IG.135779	PESCIARA	10.05	13.32	13.81	1.01	2.31	14.69	10.83	5.86	102.42	11.61	4.87	18.19	–	Lower antero-lateral	112.5
MCSNV IG.23598	PESCIARA	10.29	8.66	9.15	22.52	2.25	11.74	8.23	6.71	126.25	13.87	4.90	13.56	–	Upper lateral	144.0
MCSNV IG.43355	PESCIARA	12.26	9.01	9.53	25.24	2.26	12.36	7.83	5.88	152.41	14.85	2.58	11.59	–	Upper lateral	150.2
MCSNV IG.VR.24339	M. POSTALE	10.95	–	–	–	3.24	–	–	–	104.98	14.02	4.67	–	–	Upper antero-lateral	–
MCSNV IG.VR.24423	PESCIARA	7.48	11.26	11.81	1.09	3.00	12.41	9.32	3.43	106.31	11.16	4.51	15.77	–	Anterior	91.3
MCSNV IG.VR.66977	M. POSTALE	7.25	8.41	9.22	1.05	2.08	9.4	8.02	5.39	105.35	9.55	4.48	12.89	–	Lower antero-lateral	61.9
MCSNV IG.VR.69484	M. POSTALE	10.94	10.16	10.46	16.67	2.49	13.43	9.22	7.17	131.04	12.58	4.07	14.23	–	Upper lateral	170.6
MCSNV IG.VR.69757	M. POSTALE	–	11.10	–	–	–	–	8.67	–	–	–	–		3.59	?	–
MCSNV IG.VR.69800	M. POSTALE	5.54	9.20	9.57	1.10	2.40	9.56	7.81	3.47	86.03	6.40	2.89	12.09	–	Anterior	70.1
MCSNV T.176	PESCIARA	14.40	9.38	9.85	27.19	2.88	14.99	8.11	8.41	120.15	16.82	4.91	14.29	–	Upper lateral	156.7
MGP-PD 7358	M. POSTALE	9.05	–	–	1.20	3.33	–	–	6.31	88.95	12.53	6.88	–	–	Anterior	–
MGP-PD 7366	M. POSTALE	12.85	14.22	15.29	5.36	3.47	16.09	12.48	8.68	125.01	15.93	5.30	19.52	–	Upper antero-lateral	151.1
NHMUK PV.OR.43450	PESCIARA	7.18	10.52	10.79	1.10	2.89	11.5	9.50	4.71	103.99	9.42	4.61	15.13	–	Anterior	83.7

Abbreviations: BCT, basal crown thickness; BCW, basal crown width; CH, crown height; DCL, distal crown edge length; DS, degree of slant; LCH, height of lateral cusplets; MCL, mesial crown edge length; PCH, height of principle cusp; PCW, width of principle cusp; RA, angle between root lobes; RH, root height; RW, root width; TH, total height of tooth.

The anterior teeth (Figure 2(A)–(E)) have a relatively straight and triangular cusp, which is not very high, and regularly decreasing in width from the base to the apex. The cutting edges reach the base of the crown. Although it was not possible to detect the lateral profile in most of the specimens, MCSNV IG.VR.69757 enables this view of the cusp (Figure 2(E)) and suggests that the profile is slightly sigmoidal, as detected for some anterior teeth of *B. lerichei* by Arambourg (1952, pl. 13, figs. 17, 20), Cappetta and Nolf (2005, pl. 2, fig. 1b), and Cappetta (2012, fig. 182b). The lingual crown face of the cusp is only partially exposed in MGP-PD 7358 (Figure 2(C)) and shows a strongly convex profile. However, it is not possible to detect the tiny lingual folds usually present in anterior teeth of *Brachycarcharias*, although this condition can be explained, at least in part, by the general trend in which the folds gradually reduce and finally disappear from Danian to Lutetian specimens (Cappetta and Nolf 2005). The labial crown face of the cusp in MCSNV IG.VR.69800 (Figure 2(A)) overhangs the root by a little protruding bead, and is characterised by the presence of very short and slender folds. There is a pair of high and upright cusplets, well-separated from the main cusp. The root is high with rather long and separated lobes producing a broad and concave basal edge outline that has an angle ranging from 86° to 106°. The mesial lobe of the root appears slightly narrower and longer than the distal one.

The cusp of lower antero-lateral teeth is upright and symmetrical (Figure 2(F)–(H)) resembling the teeth figured by Cappetta and Nolf (2005, pl. 2, figures 2–6). The antero-lateral teeth bear a single pair of straight lateral cusplets, slightly lower than those of the anterior teeth. The holaulacorhize root possesses lobes, which are more divergent (producing an angle always larger than 100°), and with flatter lateral ends with respect to those of anterior teeth. The nutritive axial furrow on the lingual protuberance is deep and strong with a marked nutritive foramen (Figure 2(H)).

In upper antero-lateral teeth (Figure 3(A)–(C)) the main cusp is lower and distally inclined compared to the anterior and lower antero-lateral ones [degree of slant (DS) between 5° and 6°]. The distal cutting edge is slightly concave, whereas the mesial one is straight. A weak depression is recognisable at the base of the crown in labial view in MGP-PD 7366 (Figure 3(A_1_)), whereas small, tiny, folds are present in lingual view (Figure 3(A_2_)). The proximal lateral cusplets become broader, and a smaller second pair of cusplets can be recognised at least partially in MC 89 (Figure 3(B)).

Upper teeth of more lateral position (Figure 3(D)–(G)) have a triangular cusp, which is strongly bent distally (DS ranging between 17° and 27°). The cusp appears slightly labio-lingually flattened. The labial face of crown is smooth, almost flat with a shallow median concavity at base (Figure 3(E)–(F)). The lingual face is gently convex and there are no lingual folds on the crown (Figure 3(D) and (G)), as diagnosed for upper lateral teeth of *B. lerichei* by Cappetta and Nolf (2005). The cutting edge extends for the full height of crown. The mesial cutting edge of the main cusp is oblique and slightly convex, whereas the distal one is concave. The labial bulge of the crown is wide and overhangs the root. There are two pairs of lateral cusplets: the proximal ones are broad and triangular, whereas the distal ones are significantly reduced. However, some of the lateral teeth have a single pair of cusplets, resembling the condition of some of the lateral teeth of *B. lerichei* as noted by Cappetta and Nolf (2005). The cutting edges of the principal proximal cusplets are separated from those of the main cusp by a distinct notch. The root is low and broad, with lobes well-separated by a wide angle (RA ranging from 120° to 152°). The lingual furrow on the protuberance is weak or absent.

### Biometric remarks and body size estimation

**Figure 4.**
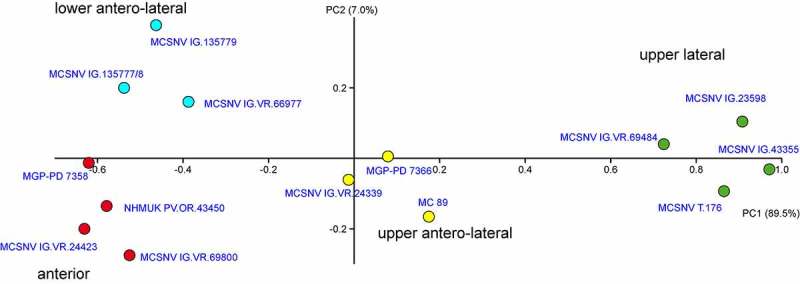
Visual image of the PCA performed on the entire data-set of standardised and log-transformed measurements, showing the separation of the teeth of *Brachycarcharias lerichei* (Casier, 1946) from the Eocene Bolca Lagerstätte based on their position on jaws. Note that the specimen MCSNV IG.VR.69757 is not present in the plot because of the lack of several measurements useful to define its position.

The PCA performed on the entire data-set of standardised and log-transformed measurements produced 10 PC axes, with the first two explaining 96.5% of the variation (Figure 4). The component loading values of the PCs were used to interpret the ‘meaning’ of the components (Hammer et al. 2001). The symmetry of the main cusp is the main character to discriminate teeth in three groups: anterior and antero-laterals, having a DS of almost 0°, upper antero-laterals (5–6° DS), and upper laterals (16–27° DS). Anterior and anterolateral teeth can be additionally distinguishable based on the principal cusp width (PCW) and root height (RH) along PC2.

The PC1 (89.5% of variance) is related to the inclination of the main cusp (DS): positive scores of the PC1 are related to strongly bent upper anterior teeth, whereas teeth having an upright cusp with almost 0° or low inclination displaying negative values. PC2 (7.0% of total variance) is mostly related to PCW and RH, but the axis appears to be only useful to separate anterior teeth (negative PC2 scores, related to low PCW and high RH) from antero-lateral teeth (positive PC2 scores, related to higher PCW and lower RH). The morphospace built on the first two PCs shows therefore a remarkable separation of the groups (Figure 4) and the PERMANOVA indicates that all clusters are significantly separated (*p* < 0.05), except for a single pairwise comparison between lower antero-lateral and upper antero-lateral teeth (*p* > 0.05). This indicates that tooth measurements are useful, at least in part, to distinguish the teeth based on their jaw position in *Brachycarcharias lerichei*.

**Figure 5.**
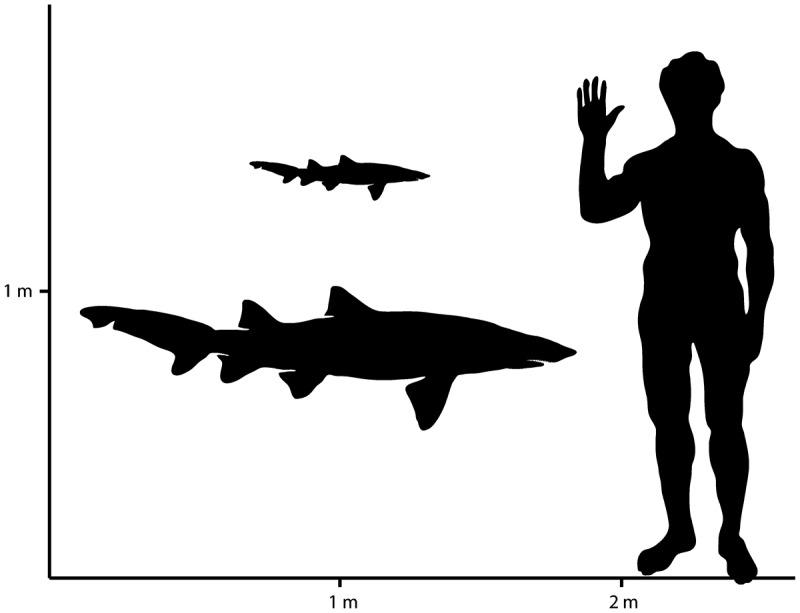
Hypothetical outlines of individuals of *Brachycarcharias lerichei* (Casier, 1946) from the Eocene Bolca Lagerstätte, showing the range of maximum and minimum body size estimation with a human for scale.

Using the procedure of Shimada (2005) for estimating the body size of *B. lerichei* (see Table 1), the crown height (CH) of the anterior and lower antero-lateral teeth ranges between 8.4 and 13.3 mm resulting in a total body size between 62 and 113 cm. The total body length, estimated based on the upper antero-lateral teeth (10.9–14.2 mm CH), ranges between 109 and 151 cm. Finally, considering the upper more lateral teeth (8.7–10.2 mm CH), the body size estimation ranges between 144 and 171 cm. Based on the entire sample we can therefore suppose the presence of several individuals in the Pesciara and Monte Postale palaeobiotopes having a total size ranging between 62 and 171 cm (Figure 5). Considering that the largest tooth found in Bolca deposit is an upper antero-lateral tooth of 19.52 mm TH (CH = 14.22 mm; TL = 151.1 cm), and that the largest tooth size for *B. lerichei* is about 25 mm TH (Cappetta 2012), it is highly likely that the maximum size of an adult individual of *Brachycarcharias lerichei* was around 200 cm TL [easily obtainable with a simple calculation: (151.1 × 25)/19.52 = 193.5]. In this perspective, the presence of individuals ranging between 62 and 171 cm might suggest that *B. lerichei* was likely represented by juvenile and adult individuals in the Bolca palaeobiotopes. There are no significant differences in the mean body size between the two populations from Pesciara and Monte Postale sites (Student’s *t* = −0.37; *p* = 0.72), probably due to the small sample considered.

## Discussion

### Comparisons

The analysis of the tooth morphology of the material from the Pesciara and Monte Postale sites has revealed the presence of several characters that unquestionably support its assignment to the lamniform family Odontaspididae, including a strong monognathic heterodonty, a tearing type dentition, well-developed and separated root lobes, a marked nutritive furrow, and a tall and slender main cusp (Compagno 1984, 1999; Cappetta 2012).

Teeth from the Bolca Konservat-Lagerstätte can be distinguished from those of other Eocene odontaspidids by their unique combination of characters such as a triangular, not very high cusp, which decreases regularly in width distally. For this reason they can be easily separated from those of *Jaekelotodus* (wide and bulky cusp), *Odontaspis* and *Carcharias* (very sharp and high cusp), and *Hypotodus* (robust cusp) (see Cappetta and Nolf 2005; Cappetta 2012). The presence of fine folds on the labial face of the main cusp allows distinguishing them from teeth of *Araloselachus*, *Glueckmanotodus*, *Odontaspis*, *Orpodon*, and *Palaeohypotodus*, in which the crown is always completely smooth. The cutting edges in *B. lerichei* extend for the full height of crown, which separates the Bolca teeth from those of *Araloselachus*, *Carcharias*, *Hypotodus*, and *Odontaspis* in which the cutting edges do not reach the base of the crown (Cappetta and Nolf 2005; Mannering and Hiller 2008; Cappetta 2012). Although the cutting edges reach the base in *Jaekelotodus* and *Sylvestrilamia*, the teeth of *Jaekelotodus* are larger (up to 45 mm high) and have a wide and bulky main cusp almost of the same height of the root, whereas *Sylvestrilamia* possesses a main cusp with a more flexuous profile and always bearing a single pair of small lateral cusplets, which also characterises teeth of *Hypotodus* (Cappetta and Nolf 2005; Cappetta 2012). The teeth from Pesciara and Monte Postale can be easily separated from those of *Araloselachus*, *Borealotodus*, *Carcharias*, *Glueckmanotodus*, *Hypotodus*, *Jaekelotodus*, *Mennerotodus*, *Sylvestrilamia*, *Tethylamna*, and *Turania* by the presence of well-developed lateral cusplets (Cappetta and Nolf 2005; Cappetta 2012; Cappetta and Case 2016). Tall lateral cusplets also occur in *Odontaspis*, *Orpodon* and *Palaeohypotodus*. However, contrary to *Brachycarcharias*, teeth of *Orpodon* are much smaller (less than 12 mm), *Odontaspis* possess a very high, sharp and not sigmoidal main cusp often flanked by three pairs of cusplets on lateral teeth, and *Palaeohypotodus* possesses short and strong folds on the labial bulge, and strong and irregular serrations at base of the cutting edges (Compagno 1984; Cappetta 2012). Finally, the stratigraphic age of the teeth provides some additional information for separating the Bolca teeth described here from *Araloselachus*, *Orpodon*, and *Tethylamna* since these genera are unknown in Ypresian deposits (see Glikman 1964; Ward and Wiest 1990; Cappetta and Nolf 2005; Cappetta 2012; Cappetta and Case 2016).

Within the genus *Brachycarcharias*, three species other than *B. lerichei* are currently recognised (*B. atlasi*, *B. koerti*, and *B. mississippiensis*). Isolate teeth from the Thanetian to the Ypresian of Northern Africa referred to *Odontaspis atlasi* by Arambourg (1952) have been reassigned to *Brachycarcharias* by Cappetta and Nolf (2005). Isolated teeth of Lutetian to Priabonian age of North Africa, North America, and Asia traditionally assigned to *Otodus koerti* (Stromer, 1910) and to *Lamna* or *Cretolamna twiggsensis* (Case, 1981) have been tentatively referred to *Brachycarcharias* by Underwood et al. (2011). However, the validity of *B. twiggsensis* was later questioned by Adnet et al. (2011) and Cappetta and Case (2016) recently reassigned this species to *Tethylamna*, a genus erected for material recovered from the Lutetian of Alabama. Although Cappetta (2006) considered *B. mississippiensis* from the Thanetian to the Ypresian of North America (see also Case 1994) to be a junior synonymy of *B. lerichei*, Case et al. (2015) consider *B. mississippiensis* as a valid species. Finally, *Carcharias borodini* and *C*. *hynei* from the Thanetian to Ypresian of Mississippi (Case 1994) are considered to be junior synonyms of *B*. *lerichei* by Cappetta (2006). Considering *B. atlasi*, *B. koerti*, *B. lerichei*, and *B. mississippiensis* to be the only valid species of *Brachycarcharias*, we can easily exclude the teeth from the Pesciara and Monte Postale as belonging to *B. atlasi*, because this species is characterised by a flat lingual crown face bearing strong folds and a well-developed second pair of lateral cusplets (Arambourg 1952), whereas *B. lerichei* possesses a strongly convex lingual face with faint or absent folds, and an extremely reduced or absent second pair of lateral cusplets (Arambourg 1952; Cappetta and Nolf 2005). The Bolca teeth can be also distinguished from those of *B. koerti* since the latter has lower lateral cusplets, particularly broad in lateral teeth and hook-shaped in the antero-lateral ones (see Dartevelle and Casier 1943; Underwood et al. 2011). Finally, although the teeth of *B. lerichei* are morphologically very similar to those of *B. mississippiensis*, we can exclude that the teeth pertain to this species mostly for biogeographic reasons as it was restricted to the eastern coast of North America (Case 1994; Case et al. 2015; see next paragraph), and the fact that the Bolca lies in an area of high number of *B. lerichei* occurrences in the Ypresian Tethys.

### Palaeobiogeography, palaeobiology and evolutionary significance

The occurrence of *B. lerichei* in the Eocene tropical reef-associated Bolca Lagerstätte provides new insights into the distribution, palaeobiology, and diversity of *Brachycarcharias* during the early Palaeogene.

**Figure 6.**
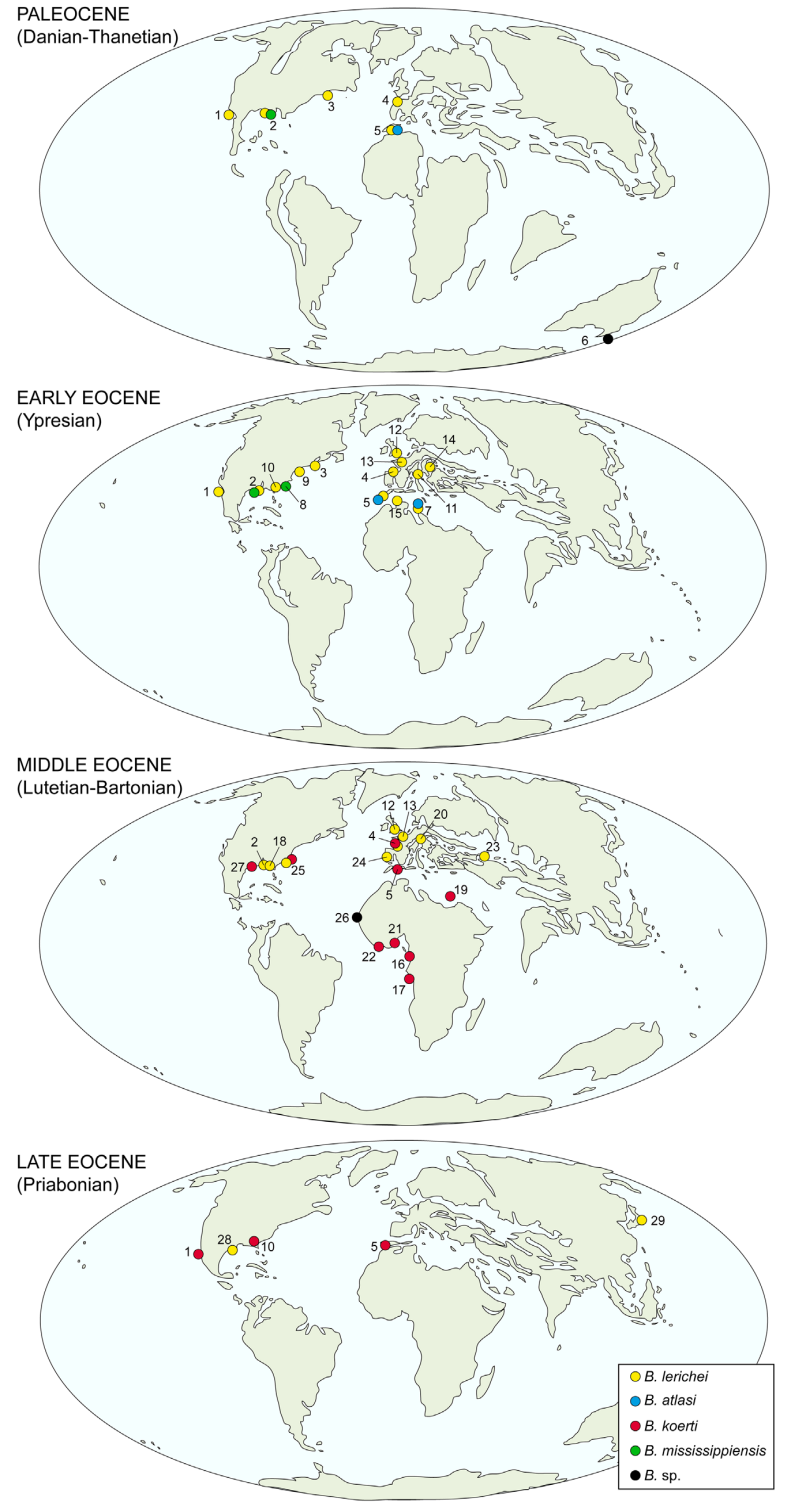
Schematic simplified map showing the palaeobiogeographic distribution of *Brachycarcharias* Cappetta and Nolf, 2005. Localities: 1 Mexico, 2 Mississippi, 3 Maryland, 4 France, 5 Morocco, 6 New Zealand, 7 Tunisia, 8 South Carolina, 9 Virginia, 10 Georgia, 11 Italy, 12 England, 13 Belgium, 14 Austria, 15 Algeria, 16 Congo, 17 Angola, 18 Alabama, 19 Egypt, 20 Germany, 21 Nigeria, 22 Togo, 23 Uzbekistan, 24 Spain, 25 North Carolina, 26 Senegal, 27 Texas, 28 Louisiana, 29 Japan. See Supplementary material for the references. Modified from Scotese (2002).

**Figure 7.**
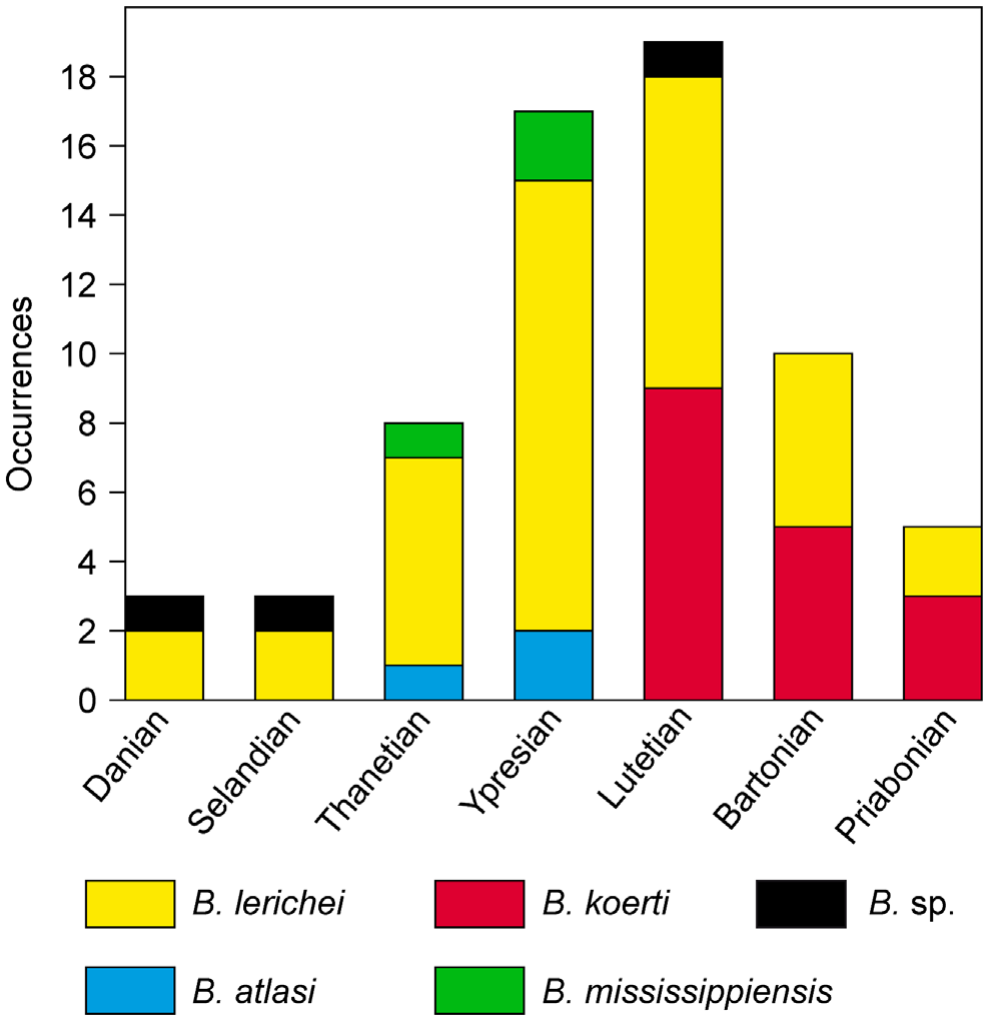
Frequency-histogram showing the distribution of the occurrences of the different species of *Brachycarcharias* Cappetta and Nolf, 2005 during the Palaeocene and Eocene.

This genus was widely distributed from the early Palaeocene (Danian) to the late Eocene (Priabonian) in the Northern Hemisphere (Figures 6 and 7). Mannering and Hiller (2008) reported *Brachycarcharias* (as *B*. sp.) in the Southern Hemisphere (New Zealand) as early as the Palaeocene, but it is only during the Middle Eocene that this genus reached its maximum geographic distribution. Teeth recovered from the Miocene of North Carolina and previously referred to *B.* sp. by Chandler (2015) have been recently assigned to *Megalolamna paradoxodon* by Shimada et al. (2017), therefore excluding the possibility of a Neogene occurrence of *Brachycarcharias*.

*B. lerichei* is the oldest and long-lived species of the genus, encompassing about 32 Ma from Danian of Maryland (Ward and Wiest 1990) and Morocco (Arambourg 1952; Noubhani and Cappetta 1997; Cappetta 2012) to the Priabonian of Louisiana (Breard and Stringer 1995) and Japan (Tanaka et al. 2006), becoming the predominant *Brachycarcharias* species of North America and the Tethys realm during the Ypresian (see Casier 1946; Arambourg 1952; Casier and Stinton 1966; Ward and Wiest 1990; Case 1994, 1967; Kent 1999; González-Barba and Thies 2000; Cappetta and Nolf 2005; Adnet and Cappetta 2008; Rayner et al. 2009; Iserbyt and De Schutter 2012; Cicimurri and Ebersole 2015). The decrease in the occurrence of *B. lerichei* at the beginning of the Lutetian coincides with the first appearance of *B. koerti* which rapidly spread to in North America, Europe, North and Western Africa up to the Priabonian (see Stromer 1910; White 1926; Dartevelle and Casier 1943, 1959; Arambourg 1952; Casier 1957; Antunes 1964; Cappetta and Traverse 1988; Dutheil 1991; Dutheil and Merle 1992; González-Barba 2003; Parmley et al. 2003; Robb 2006; Timmerman and Chandler 2008; Adnet et al. 2010; Underwood et al. 2011). The species *B. atlasi* and *B. mississippiensis* conversely appear to be restricted only to the Thanetian-Ypresian of North Africa (Arambourg 1952; Cappetta 2012) and North America (Case 1994; Case et al. 2015), respectively.

From an evolutionary point of view, the turnovers of the *Brachycarcharias* species during the early Palaeogene appear to be associated to certain morphological changes. For example, Cappetta and Nolf (2005) highlighted an increase in overall size, and a gradual disappearance of the folds on the lingual face of the crown from Danian to Lutetian. Moreover, it also was noted that this genus developed dental simplification during its evolutionary history, with a reduction of the number of lateral cusplets from the Danian *B. atlasi* to the Ypresian and Lutetian *B. lerichei* and an indeterminate species (? *B. koerti*) from the Lutetian of western Africa (Cappetta and Nolf 2005; Cappetta 2012; Cappetta and Case 2016). Finally, the dentition, usually of tearing-type in all species, tends to become of cutting-type in lateral teeth in *B. koerti* (as ‘*Carcharias*’ in Cappetta 2012). These hypotheses appear to be consistent with the very weak or absent folds, and the reduced or absent second pair of lateral cusplets observed in the Ypresian material from Bolca.

According to Maisch et al. (2014) *Brachycarcharias lerichei* may have had similar feeding and habitat preferences as the living porbeagle shark *Lamna nasus* because of their apparently similar tooth morphologies. However, *L. nasus* is a pelagic or epipelagic lamnid shark that is known to inhabit coastal temperate to cool waters on continental shelves, but also was found far offshore in ocean basins and occasionally close inshore, from the North Atlantic to temperate waters of the Southern Hemisphere, but not in equatorial tropical seas (Compagno 1984; Compagno et al. 2005; Froese and Pauly 2017). *Brachycarcharias lerichei* is the only shark, and more generally, the sole elasmobranch fish from the Bolca Lagerstätte represented uniquely by isolated teeth, contrary to the carcharhiniforms *Galeorhinus cuvieri* and *Eogaleus bolcensis*, known by several completely articulated skeletons (Cappetta 1975; Fanti et al. 2016). Based on the high-quality preservation of specimens recovered from Bolca, we hypothesise that the unique presence of isolated teeth of *B. lerichei* may reflect a real biological and ecological signal, rather than being the product of collection or taphonomic biases, suggesting that *Brachycarcharias*, contrary to the Bolca carcharhiniforms, was probably an adventitious visitor of the tropical reef-associated Bolca palaeobiotopes. In this contexts, sand/sea-grass beds, open sea, lagoon, and coral reefs concurred to create heterogeneous habitats in which zooplanktivorous fishes (mostly clupeoids) represented one of the main trophic resource for most predators (see e.g. Landini and Sorbini 1996; Marramà et al. 2016a), possibly including *B. lerichei*. Contrary to Maisch et al. (2014), it is therefore likely that *B. lerichei*, whose teeth were abundantly recovered from tropical shallow to deep water deposits (see e.g. Arambourg 1952; Ward and Wiest 1990; Case 1994; Noubhani and Cappetta 1997; Smith et al. 1999; González-Barba and Thies 2000; Cappetta and Nolf 2005; Adnet and Cappetta 2008; Diedrich 2012; Cicimurri and Ebersole 2015; Cappetta and Case 2016) was an opportunistic Palaeogene top predator with a wide range of feeding and habitat preferences.

It is also remarkable that the range of body sizes of the *B. lerichei* individuals (62–171 cm) from the Pesciara and Monte Postale sites is consistent with the relatively small size of the other Bolca selachians. The triakid *Galeorhinus cuvieri* is known by several complete articulated specimens ranging from 54 to 92 cm (all of them supposed to be juvenile individuals; Fanti et al. 2016), whereas the largest carcharhinid *Eogaleus bolcensis* is a specimen of about 138 cm (Cappetta 1975). The presence of small shark species (e.g. *B. lerichei*) and juvenile individuals (e.g. *G. cuvieri*) in the Bolca palaeobiotopes might be related, at least in part, to the competitive advantage of juveniles and small shark species (see also Motta 2004) in having access to relatively competitor-free trophic niches and food resources in the shallow water Bolca palaeobiotopes that were probably unavailable for larger top predators. This can also explain, at least in part, the exclusive presence of juvenile individuals of the triakid species *Galeorhinus cuvieri* in the Pesciara setting, contrary to the hypothesis of the presence of a nursery area proposed by Fanti et al. (2016). Our hypothesis also is supported by the fact that none of the criteria to recognise a nursery area (i.e. sharks are more commonly encountered in the area than other areas and have a tendency to remain or return for extended periods; the area is repeatedly used across years; presence of egg cases or/and gravid females; shark teeth are very common and depending on the variability of size among teeth collected for a given species in a specific locality; see Castro 1993; Heupel et al. 2007; Pimiento et al. 2010; Fischer et al. 2011; Sallan and Coates 2014) can be unquestionably tested for *G. cuvieri* and, more generally, for any of the selachians of the Pesciara and Monte Postale sites.

It is has been broadly documented that the early Palaeogene was marked by high origination and turnover rates of bony and cartilaginous fish lineages that were related to the opportunistic ecological niche-filling scenario after the K-Pg boundary event in pelagic and benthic environments (Walker and Brett 2002; Kriwet and Benton 2004; Friedman 2009, 2010; Guinot and Cavin 2016; Marramà et al. 2016b, 2016c). Most of the medium to large-sized, fast-swimming top predators of epipelagic and shelf zones having a rather broad-spectrum diet as *Archaeolamna*, *Paranomotodon*, *Squalicorax*, *Cretoxyrhina*, and *Scapanorhynchus* disappeared at the end of the Cretaceous (Kriwet and Benton 2004). It also has been suggested that, starting in the Danian, these top predators were replaced by other pelagic selachians with similar adaptations as carcharhinids and isurids (Kriwet and Benton 2004; Friedman and Sallan 2012). In this perspective, the Danian rise and diversification of *Brachycarcharias* might be considered as another example of opportunistic ecological replacement in high trophic levels experimented by the Odontaspididae in the context of the massive adaptive fish radiation in the aftermath of the end-Cretaceous extinction. The diversity and co-existence of several odontaspidid taxa in the Eocene (at least seven genera and 12 species in the Ypresian; see Cappetta 2012), support the hypothesis that the representatives of this family were more diverse than today (with *Carcharias taurus*, *Odontaspis ferox and O. noronhai* as the only extant representative of the family), and that the present reduced taxonomic diversity of the Odontaspididae might represent a recent phenomenon, as hypothesised for other modern selachians (e.g. *Galeorhinus*; see Adnet and Cappetta 2008).

The Priabonian (Late Eocene) marks the last occurrences of *Brachycarcharias*. The Eocene-Oligocene transition (ca. 33.7 Ma), that was characterised by declining atmospheric CO_2_ contents, a long-term deep-sea cooling, and establishment of large Antarctic ice sheets, resulted in one of the most dramatic climatic shifts of the Cenozoic, with major biotic turnovers in marine and terrestrial faunas and floras (Zachos et al. 2001; Prothero et al. 2003; Pagani et al. 2005; Lear et al. 2008; Pearson et al. 2008). In this perspective, the gradual disappearance and extinction of *Brachycarcharias* as well as other odontaspidids such as *Striatolamia* and *Palaeohypotodus* at the end of the Eocene might be related, at least in part, to a second wave of biotic turnovers that took place at the E-O boundary and that involved particularly marine top predators (Prothero et al. 2003). This turnover of taxa at the transition from the Eocene to the Oligocene was the most severe extinction event in the Cenozoic related to significant temperature declines (e.g. Zachos et al. 2001; Liu et al. 2009) and sea level falls (Harnik et al. 2012). We hypothesise that Eocene odontaspidids were partly replaced by other odontaspidids in the Oligocene (e.g. *Araloselachus*) and predominantly by lamnids (e.g. *Carcharoides*, *Isurus*, *Lethenia*) that might have been better adapted to cooler environments. This, however, needs to be tested in the future employing robust analytical approaches.

## Conclusions

A re-examination of elasmobranch remains from the famous Ypresian Bolca Lagerstätte of northern Italy revealed the first documented occurrence of *Brachycarcharias lerichei* (Casier, 1946) from this locality and allowed us to analyse the palaeobiology and palaeobiogeography of this Palaeogene sand tiger shark. The presence of several *B. lerichei* individuals of different ontogenetic stages represented by isolated teeth suggests that, contrary to the other Bolca selachians, this species was probably an opportunistic adventitious visitor of the Pesciara and Monte Postale palaeobiotopes. The diversity patterns of *Brachycarcharias* indicate that the appearance and subsequent diversification of this genus was related, at least in part, to the opportunistic niche-filling scenario in the context of the massive bony and cartilaginous adaptive radiation after the K-Pg boundary. The emergence of a new top predator in the early Palaeogene is particularly interesting, considering the coeval extensive presence of biotic turnovers (e.g. Kriwet and Benton 2004), adaptive radiations (e.g. Friedman 2009, 2010; Marramà et al. 2016b, 2016c) and rise and expansion of novel feeding strategies (e.g. piscine herbivory, high precision benthic feeding, nocturnal feeding, foraminifera feeding, ambush predation; see Goatley et al. 2010; Schmitz and Wainwright 2011; Bellwood et al. 2014; Marramà and Carnevale 2017; Marramà et al. 2017) in several bony and cartilaginous fish lineages that took place to fill the ecological roles left unoccupied by the victims of the end-Cretaceous extinction. *Brachycarcharias* was part of a recovery fauna in the aftermaths of the end-Cretaceous extinction event, which was eventually replaced by other lamniform sharks.

## Funding

The research was supported by the Austrian Federal Ministry of Science, Research and Economy [Ernst Mach grant ICM-2016-03318 to G.M.], by grants [ex-60% 2016 and 2017 to G.C.] from the Università degli Studi di Torino, and by a grant of the Austrian Science Fund (FWF) [grant number P26465-B25 to J.K].

## Disclosure statement

No potential conflict of interest was reported by the authors.

## Supplemental data

Supplemental data for this article can be accessed here http://dx.doi.org/10.1080/08912963.2017.1341503.

## Supplementary Material

Supplementary_material.docx
