# Supplementary material for: Eocene sand tiger sharks (Lamniformes, Odontaspididae) from the Bolca Konservat-Lagerstätte, Italy: palaeobiology, palaeobiogeography and evolutionary significance
Source: Hist Biol. 2017 Jun 22;31(2):102–16. doi: 10.1080/08912963.2017.1341503 (PMC6343108; doi:10.1080/08912963.2017.1341503)
Supplement: Supplementary_material.docx [file GHBI_A_1341503_SM2650.docx]

Supplementary material to the article:

**Eocene sand tiger sharks (Lamniformes, Odontaspididae) from the Bolca Konservat-Lagerstätte, Italy: Palaeobiology, palaeobiogeography and evolutionary significance**

Giuseppe Marramà^a^, Andrea Engelbrecht^a^, Giorgio Carnevale^b^ and Jürgen Kriwet^a^

^a^ *University of Vienna, Department of Palaeontology, Althanstrasse 14, 1090, Vienna, Austria; marramag81@univie.ac.at;* [*andrea.engelbrecht@univie.ac.at*](mailto:andrea.engelbrecht@univie.ac.at)*; juergen.kriwet@univie.ac.at*

^b^ *Università di Torino, Dipartimento di Scienze della Terra, via Valperga Caluso 35, 10125, Torino, Italy; giorgio.carnevale@unito.it*

Corresponding author: Giuseppe Marramà; ORCID: 0000-0002-7856-5605

**Appendix 1**. Morphometric tooth terminology adopted and modified from Kriwet et al. (2015). Abbreviations: BCT, basal crown thickness; BCW, basal crown width; CH, crown height; DCL, distal crown edge length; DS, degree of slant; LCH, height of lateral cusplets; MCL, mesial crown edge length; PCH, height of principle cusp; PCW, width of principle cusp; RA, angle between root lobes; RH, root height; RW, root width; TH, total height of tooth.

**Appendix 2.** Comprehensive list of all known occurrences of *Brachycarcharias* used to assess its palaeobiogeography and diversity patterns through time.

| **Epoch** | **Age** | **Species** | **Locality** | **References** |  |
| --- | --- | --- | --- | --- | --- |
| Palaeocene | Danian | *B. lerichei* | Maryland | Ward & Wiest 1990 |  |
|  |  |  | Morocco | Arambourg 1952; Noubhani & Cappetta 1997; Cappetta 2012; Gajić et al. 2014 |  |
|  |  | *B.* sp. | New Zealand | Mannering & Hiller 2008 |  |
|  | Selandian | *B. lerichei* | Maryland | *ghost occurrence* (present both in Danian and Thanetian strata of Maryland) |  |
|  | |  |  | Morocco | Arambourg 1952; Cappetta & Nolf 2005 |
|  |  | *B.* sp. | New Zealand | Mannering & Hiller 2008 |  |
|  | Thanetian | *B. atlasi* | Morocco | Arambourg 1952 |  |
|  |  | *B. lerichei* | France | Smith et al. 1999 |  |
|  |  |  | Maryland | Ward & Wiest 1990 |  |
|  |  |  | Mexico | González-Barba & Thies 2000 |  |
|  |  |  | Mississippi | Case 1994; Cappetta 2006 |  |
|  |  |  | Morocco | Noubhani & Cappetta 1997; Cappetta & Nolf 2005; Cappetta 2012 |  |
|  |  | *B. mississippiensis* | Mississippi | Case 1994; Case et al. 2015 |  |
| Eocene | Ypresian | *B. atlasi* | Morocco | White 1934; Arambourg 1952; Cappetta 2006 |  |
|  |  |  | Tunisia | Arambourg 1952 |  |
|  |  | *B. lerichei* | Algeria | Arambourg 1952 |  |
|  |  |  | Austria | Schultz & Piller 2013 |  |
|  |  |  | Belgium | Casier 1946; Cappetta & Nolf 2005; Iserbyt & De Shutter 2012 |  |
|  |  |  | England | Woodward 1889; Arambourg 1952; Casier & Stinton 1966; Rayner et al. 2009 |  |
|  |  |  | France | Adnet & Cappetta 2008 |  |
|  |  |  | Georgia | Cicimurri & Ebersole 2015 |  |
|  |  |  | **Italy** | **Bassani 1897; this paper** |  |
|  |  |  | Maryland | Ward & Wiest 1990 |  |
|  |  |  | Mexico | González-Barba & Thies 2000 |  |
|  |  |  | Mississippi | Case 1994; Cappetta 2006 |  |
|  |  |  | Morocco | Arambourg 1952 |  |
|  |  |  | Tunisia | Arambourg 1952 |  |
|  |  |  | Virginia | Case 1967; Kent 1999 |  |
|  |  | *B. mississippiensis* | Mississippi | Case 1994; Case et al. 2015 |  |
|  |  |  | South Carolina | Case et al. 2015 |  |
|  | Lutetian | *B. koerti* | Angola | Dartevelle & Casier 1943; Casier 1957; Antunes 1964 |  |
|  |  |  | Congo | Dartevelle & Casier 1943, 1959 |  |
|  |  |  | Egypt | Underwood et al. 2011 |  |
|  |  |  | France | Dutheil 1991; Dutheil & Merle 1992 |  |
|  |  |  | Morocco | Arambourg 1952; Adnet et al. 2010 |  |
|  |  |  | Nigeria | White 1926 |  |
|  |  |  | North Carolina | Timmerman & Chandler 2008 |  |
|  |  |  | Texas | Robb 2006 |  |
|  |  |  | Togo | Stromer 1910; Arambourg 1952; Cappetta & Traverse 1988 |  |
|  |  | *B. lerichei* | Alabama | Maisch et al. 2014; Cappetta & Case 2016; |  |
|  |  |  | Belgium | Arambourg 1952; Cappetta & Nolf 2005; Van Den Eeckhaut & De Shutter 2009 |  |
|  |  |  | England | Arambourg 1952 |  |
|  |  |  | France | Arambourg 1952 |  |
|  |  |  | Germany | Van Der Hocht 1979; Diedrich 2012 |  |
|  |  |  | Mississippi | Cappetta 2012 |  |
|  |  |  | North Carolina | Case & Borodin 2000; Timmerman & Chandler 2008; |  |
|  |  |  | Spain | Bauzá & Gomez Pallerola 1988 |  |
|  |  |  | Togo | Stromer 1910; Cappetta & Nolf 2005 |  |
|  |  | *B.* sp. | Senegal | Siguendibo Sambou et al. 2017 |  |
|  | Bartonian | *B. koerti* | Congo | Dartevelle & Casier 1943, 1959 |  |
|  |  |  | Egypt | Underwood et al. 2011 |  |
|  |  |  | Morocco | Adnet et al. 2010 |  |
|  |  |  | North Carolina | Timmerman & Chandler 2008 |  |
|  |  |  | Texas | Robb 2006 |  |
|  |  | *B. lerichei* | Belgium | Arambourg 1952 |  |
|  |  |  | England | Arambourg 1952 |  |
|  |  |  | North Carolina | Case & Borodin 2000; Timmerman & Chandler 2008; |  |
|  |  |  | Togo | Stromer 1910 |  |
|  |  |  | Uzbekistan | Case et al. 1996; Malyshkina & Ward 2016 |  |
|  | Priabonian | *B. koerti* | Georgia | Parmley et al. 2003 |  |
|  |  |  | Mexico | González-Barba 2003 |  |
|  |  |  | Morocco | Gajić et al. 2014 |  |
|  |  | *B. lerichei* | Japan | Tanaka et al. 2006 |  |
|  |  |  | Louisiana | Breard & Stringer 1995 |  |

**Supplementary references**

Adnet S, Cappetta H. 2008. New fossil triakid sharks from the early Eocene of Prémontré, France, and comments on fossil record of the family. Acta Palaeontol Pol. 53:433–448.

Adnet S., Cappetta H., Tabuce R. 2010. A Middle-Late Eocene vertebrate fauna (marine fish and mammals) from southwestern Morocco; preliminary report: age and palaeobiogeographical implications. Geol Mag. 147:860–870.

Antunes MT. 1964. Neocretácio e o Cenozóico do Litoral de Angola [Neo-Cretaceous and Cenozoic of the Angola litoral]. Lisboa: Junta Invest. Ultramar.

Arambourg C. 1952. Les vertébrés fossiles des gisements de phosphates (Maroc-Algeérie-Tunisie) [Fossil vertebrates from the phosphatic sites of Morocco, Algerie and Tunisia]. Notes Mém Serv Géol Maroc. 92:1–372.

Bassani F. 1897. Aggiunte all’ittiofauna Eocenica dei Monti Bolca e Postale [Additions to the Eocene ichthyofauna of Monte Bolca and Postale]. Palaeontogr Ital. 3:77–88.

Bauzá RJ, Gomez Pallerola JE. 1988. Contribución al conocimiento de la ictiología fósil de España [Contribution to the knowledge of the fossil ichthyology of Spain. Boll Soc Hist Nat Balears. 32:115–138

Breard S, Stringer G. 1995. Paleoenvironment of a diverse marine vertebrate fauna from the Yazoo Clay (Late Eocene) at Copenhagen, Caldwell Parish, Louisiana. Trans GCAG. 45:77–85.

Cappetta H. 2006. Elasmobranchii post-Triadici (index generum et specierum). In: Riegraf W, editor. Fossilium Catalogus I: Animalia. Leiden: Backhuys Publishers; p. 1–472.

Cappetta H. 2012. Handbook of Paleoichthyology - Chondrichthyes - Mesozoic and Cenozoic Elasmobranchii: Teeth. München: Verlag Dr. Friedrich Pfeil.

Cappetta H, Case G. 2016. A selachian fauna from the Middle Eocene (Lutetian, Lisbon Formation) of Andalusia, Covington County, Alabama, USA. Palaeontogr Abt A. 307(1-6):43–103.

Cappetta H, Nolf D. 2005. Revision of some Odontaspididae (Neoselachii: Lamniformes) from the Paleocene and Eocene of the North Sea Basin. Bull Inst R Sci Nat Bel. 75:237–266.

Cappetta H, Traverse M. 1988. Une riche faune de sélaciens dans le bassin à phosphate de Kpogamé-Hahotoé (Éocène moyen du Togo): Note préliminaire et précisions sur la structure et l'âge du gisement [A rich selachian fauna from the phosphatic basin of Kpogamé-Hahotoé (Middle Eocene of Togo): Preliminaries notes and clarification on the structure and age of the deposit]. Géobios 21(3):359–365. doi: 10.1016/S0016-6995(88)80058-5

Case GR. 1967. Eocene fossils of the Aquia Formation (Virginia). Earth Sci Mag. 20(5):211–214.

Case GR. 1994. Fossil fish remains from the Late Paleocene Tuscahoma and Early Eocene Bashi Formations of Meridian, Lauderdale County, Mississippi - Part I. Selachians. - Palaeontogr Abt A. 230(4-6):97–138.

Case GR, Borodin PD. 2000. Late Eocene selachians from Irwinton Sand Member of the Barnwell Formation (Jacksonian), WKA mines, Gordon, Wilkinson Country, Georgia. Münchner Geowiss. Abh. A. 39:5–16

Case GR, Cook TD, Wilson MVH. 2015. A new elasmobranch assemblage from the early Eocene (Ypresian) Fishburne Formation of Berkeley County, South Carolina, USA. Can J Earth Sci. 52:1–16.

Case GR, Udovichenko NI, Nessov LA, Averianov AO, Borodin PD. 1996. A Middle Eocene selachian fauna from the White Mountain formation of the Kizylkum desert, Uzbekistan, C.I.S. Palaeontogr Abt. A. 242:99–126

Casier E. 1946. La faune ichthyologique de l'Yprésien de la Belgique [The ichthyofauna of the Ypresian of Belgium]. Mém Mus R Hist Nat Belg. 104:1–267.

Casier E. 1957. Les faunes ichthyologiques du Crétacé et du Cénozoïque de l'Angola et de l'Enclave de Cabinda. Leurs affinités paléobiogéographiques [Ichyhofaunas of the Cretaceous and Cenozoic of Angola and Englave of Cabinda. Their palaeobiogeographic affinities]. Com Serv Geol Portugal. 38(2):269–290.

Casier E, Stinton FC. 1966. Faune ichthyologique du London Clay [Ichthyological fauna of London Clay]. London: Trustees of the British Museum.

Cicimurri DJ, Ebersole JA. 2015. Two new species of *Pseudaetobatus* Cappetta, 1986 (Batoidei: Myliobatidae) from the southeastern United States. Palaeontol Electron. 18.1.15A:1–17.

Dartevelle E, Casier E. 1943. Les poissons fossiles du Bas-Congo et des regions voisines [The fossil fishes of Bas-Congo and nearby regions]. Ann Mus Congo Belg Ser A (Miner Geol Paleontol). 2(1):1–200.

Dartevelle E, Casier E. 1959. Les poissons fossiles du Bas-Congo et des régions voisines [The fossil fishes of Bas-Congo and nearby regions]. Mus Congo Belg Ser A (Miner Geol Paleontol). 2(3): 257–568.

Diedrich CG. 2012. Eocene (Lutetian) Shark-rich coastal paleoenvironments of the Southern North Sea Basin in Europe: Biodiversity of the marine Fürstenau Formation Including Early white and megatooth sharks. Int J Oceanogr. 2012:565326. doi: 10.1155/2012/565326

Dutheil DB. 1991. A checklist of Neoselachii (Pisces, Chondrichthyes) from the Palaeogene of the Paris Basin, France. Tertiary Res. 13(1):27–36.

Dutheil DB, Merle D. 1992. Les chondrichthiens elasmobranches del la Tuilerie de Gan (Pyrénées Atlantiques, France) [The elasmobranch chondrichthyans from the Tuileriede Gan (Atlantic Pyrenees, France)]. Cossmanniana. 1(2–4):15–26.

Gajić A, Hanjalić J, Davidov B. 2014. Frequency, taxonomy and morphology of different shark taxa of Lower Paleogene and Upper Cretaceous from Morocco, North Africa. Pluralidade. 3(3):54–68.

González-Barba G. 2003. Descripción de asociaciones faunísticas de elasmobranquios fósiles del eoceno superior (Priaboniano) de las formaciones Tepetate y Bateque de Baja California Sur, México [Description of the faunal association of the fossil elasmobranchs of the Late Eocene (Priabonian) of the Tepetate and Bateque de Southern Baja California Formations, Mexico] [dissertation]. La Paz: Centro Interdisciplinario de Ciencias Marinas.

González-Barba G, Thies D. 2000. Asociaciones faunisticas de condrictios en el Cenozoico de la Peninsula de Baja California, Mexico [Faunal associations of the chondrichthyans from Cenozoic of the Peninsula of Baja California, Mexico]. Profil, 18:1–4.

Iserbyt A, De Schutter PJ. 2012. Quantitative analysis of Elasmobranch assemblages from two successive Ypresian (early Eocene) facies at Marke, western Belgium. Geol Belg. 15(3):146–153.

Kent BW. 1999. Sharks from the Fisher/Sullivan site. In: Weems RE, editor. Fossil vertebrates and plants from the Fisher/Sullivan site (Stafford County): A record of Early Eocene life in Virginia. Virginia: Virginia Division of Mineral Resources publication 192; p. 11–37.

Maisch HM, Becker MA, Raines BW, Chamberlain JA. 2014. Chondrichthyans from the Tallahatta-Lisbon Formation contact (Middle Eocene), Silas, Choctaw County, Alabama. Paludicola 9(4):183–209.

Malyshkina TP, Ward DJ. 2016. The Turanian basin in the Eocene: the new data on the fossil sharks and rays from the Kyzylkum desert (Uzbekistan). Proc Zool Inst RAS:320:50–65.

Mannering AA, Hiller N. 2008. An early Cenozoic neoselachian shark fauna from the southwest Pacific. Palaeontology. 51:1341–1365.

Noubhani A, Cappetta H. 1997. Les Orectolobiformes, Carcharhiniformes et Myliobatiformes (Elasmobranchii, Neoselachii) des bassins phosphate du Maroc (Maastrichtien-Lutetien basal). Systematique, biostratigraphie, evolution et dynamique des faunes [The Orectolobiformes, Carcharhiniformes and Myliobatiformes (Elasmobranchii, Neoselachii) of the phosphatic basins of Morocco (Maastrichtian-early Lutetian)]. Palaeo Ichthyol. 8:1–327.

Parmley D, Cicimurri DJ, Campbell B. 2003. Late Eocene sharks of the Hardie Mine local fauna of Wilkinson County, Georgia. Georgia J Sci. 61(3):153–179.

Rayner D, Mitchell T, Rayner M. 2009. London Clay fossils of Kent and Essex. Rochester: Medway Fossil and Mineral Society.

Robb AJ. 2006. Middle Eocene shark and ray fossils of Texas. The Backbender's Gazette. 2006: 9–13.

Schultz O, Piller WE. 2013. Catalogus Fossilium Austriae Band 3 – Pisces. Ein systematisches Verzeichnis aller auf österreichischem Gebiet festgestellten Fossilien [Catalogus Fossilium Austriae Volume 3 – Pisces. A systematic register of fossils found in Austria]. Austria: VoaW.

Siguendibo Sambou B, Sarr R, Hautier L, Cappetta H, Adnet S. 2017. The selachian fauna (sharks and rays) of the phosphate series of Ndendouri-Ouali Diala (Matam, Western Senegal): Dating and paleoenvironmental interests. N. Jb. Geol. Paläont. Abh. 283:205–219.

Smith R, Smith TH, Steurbaut E. 1999. Les élasmobranches de la transition Paléocène-Eocène de Dormaal (Belgique): implications biostratigraphiques et paléobiogéographiques [The elasmobranchs of the Palaeocene-Eocene transition of Dormaal (Belgium): biostratigraphic and palaeobiogeographic implications]. Bull Soc géol Fr. 170(3):327–334.

Stromer E. 1910. Reptilien und Fischreste aus dem marinen Alttertiär von Südtogo (West Africa) [Reptile and fish remains from the Early Tertiary of South Togo]. Z Dtsch Geol Ges. 62(7):478–505.

Tanaka T, Fujita Y, Morinobu S. 2006. Fossil shark teeth from the Namigata Formation in Ibara City, Okayama Prefecture, Central Japan and their biostratigraphical significance. Bull Mizunami Foss Mus. 33:103–109.

Timmerman J, Chandler RE. 2008. Cretaceous and Paleogene fossils of North Carolina, A field guide. Durham: North Carolina Fossil Club.

Underwood CJ, Ward DJ, King C, Antar SM, Zalmout IS, Gingerich PD. 2011. Shark and ray faunas in the Middle and Late Eocene of the Fayum Area, Egypt. Proc Geol Ass. 122:47–66.

Van Den Eeckhaut G, De Schutter P. 2009. The elasmobranch fauna of the Lede Sand Formation at Oosterzele (Lutetian, Middle Eocene of Belgium). Palaeofocus. 1:1–57.

Von Der Hocht F. 1979. Eine Lagerstätte kreidezeitlicher und paläogener Chondrichthyes-Reste bei Fürstenau (Niedersachsen) [A deposit of Cretaceous and Palaeogene chondrichthyan remains near Fürstenau (Lower Saxony)]. Osnabrücker Naturwissenschaftliche Mitteilungen. 6:35–44

Ward DJ, Wiest RL. 1990. A checklist of Palaeocene and Eocene sharks and rays (Chondrichthyes) from the Pamunkey Group, Maryland and Virginia, USA. Tertiary Res. 12(2):81–88.

White EI. 1926. Eocene fishes from Nigeria. Bull geol Surv Nigeria. 10:1–82.

White EI. 1934. Fossil fishes of Sokoto province. Bull geol Surv Nigeria. 14:1–78.

Woodward AS. 1899. Notes on the teeth of sharks and skates from English Eocene formations. Proc Geol Ass London. 16:1–14.
